# Supplementary material for: Transcriptome analysis reveals novel regulatory mechanisms in a genome-reduced bacterium
Source: Nucleic Acids Res. 2014 Oct 31;42(21):13254–68. doi: 10.1093/nar/gku976 (PMC4245973; doi:10.1093/nar/gku976)
Supplement: SUPPLEMENTARY DATA [file supp_42_21_13254__index.html]

Transcriptome analysis reveals novel regulatory mechanisms in a genome-reduced bacterium — Transcriptome analysis reveals novel regulatory mechanisms in a genome-reduced bacterium — SUPPLEMENTARY DATA 

# Transcriptome analysis reveals novel regulatory mechanisms in a genome-reduced bacterium

## SUPPLEMENTARY DATA

**Files in this Data Supplement:**

- SUPPLEMENTARY DATA
- SUPPLEMENTARY DATA
- SUPPLEMENTARY DATA
