# Supplementary material for: Transcriptome analysis reveals novel regulatory mechanisms in a genome-reduced bacterium
Source: Nucleic Acids Res. 2014 Oct 31;42(21):13254–68. doi: 10.1093/nar/gku976 (PMC4245973; doi:10.1093/nar/gku976)
Supplement: SUPPLEMENTARY DATA [file supp_gku976_nar-02483-x-2014-File007.pdf]

Table of contents

- Table of contents
- Supplementary Text
  - Dependence between RBS and protein abundancy
  - RNA to protein correlation during heat shock
- Supplementary Tables
  - Table S1. Sample information
  - Table S2. Real-time PCR
  - Table S3. Transcription intervals
  - Table S4. TSS position (5-ERS)
  - Table S5. Analysis of differential expression of CDS under different conditions
  - Table S6. GO-enrichment of stress-related genes
  - Table S7. GO-enrichment for clusters of heat shock related genes
  - Table S8. CIRCE matches
  - Table S9. GO-enrichment for TSS and TTs heat shock up-regulated genes regulated genes
  - Table S10. PWM of promoter sequence
  - Table S11. CDS read counts
  - Table S12. Protein abundance indexes
  - Table S13. Analysis of RNA to protein correlation within GO groups
  - Table S14. RT-PCR analysis of heat shock-related regulation of four selected hairpinless down-CS
  - Table S15. Transformation frequencies with different vector amount.
  - Table S16. Positions of vector integration sites.
  - Table S17. Transcription of tetM gene in transformants by RT-qPCR.
- Supplementary Figures
  - Figure S1. Correlation heatmap for all RNA-Seq samples
  - Figure S2. Correlation between RT-PCR- and RNA-Seq-based gene expression estimates
  - Figure S3. Correlation between RT-PCR- and RNA-Seq-based gene expression fold change estimates
  - Figure S4. Sigma factor structure
  - Figure S5. Distribution of best match of promoter PWM in 100nt upstream of TSSs
  - Figure S6. AT-content near down-CS.
  - Figure S7. Ribosome binding energy
  - Figure S8. Dependence of FDR of -10 box and extension weights
  - Figure S9. Vector design for M. gallisepticum transformation
  - Figure S10. Alignment of upstream regions of rpsP (prototype), tetM and transposase.
  - Figure S11. Logo-image of -35 box
  - Figure S12. Dependence of TSS activity on number of mismatches in minus 35 box compared with consensus sequence (TTGACA)

Supplementary Text

Dependence between RBS and protein abundancy

In addition to correlation analysis between RBS dG and protein or RNA abundance described in the main text, we analyzed contribution of length of spacer between RBS and start codon. Unfortunately, we observed only weak and insignificant correlation (rho = -0.06, pv > 0.1). Then we used liner models and ANOVA to investigate joint effect of dG and spacer. No one model (with and without interaction term, considering spacer as is or as absolute difference from optimal length (6 nt)) resulted in significant (pv < 0.05) contribution of either spacer length or interaction term.

RNA to protein correlation during heat shock

As we shown in the main text, RNA-protein correlation drops during heat shock. To rather investigate it we grouped genes according to GO-terms and checked RNA to protein correlation for all GO-terms with at least 10 genes (N=33). Two thirds (22) of gene groups exhibited drop in RNA to protein correlation. Three gene groups exhibited significant change in RNA to protein correlation (Williams's Test, pv < 0.05), for all of them correlation drops under heat shock (Supplementary table S13)

Supplementary Tables

Table S1. Sample information

see file Supplementary tables, TableS1-samples tab
all samples are uploaded to SRA under project id PRJNA243934
(http://www.ncbi.nlm.nih.gov/bioproject/243934)

Table S2. Real-time PCR

Table S2a, RT-qPCR (see file Supplementary tables, TableS2a-RT-qPCR tab):
replicate row - contains unique ID of the experiment, experiment may contain several conditions, conditions are indicated on the row below. One technical replicate per each biological replicate was used. Several genes were included twice per experiment to ensure reproducibility of the data.
Columns:
R(low) - locus tag of the homologous gene in *M. gallisepticum R(low)* genome
S6 - locus tag of *M. gallisepticum S6*
gene - gene’s name according to trivial nomenclature used for bacteria, if exists
Data in the table is provided as raw C_t aligned by 23S rRNA C_t.

Table S2b, primers (see file Supplementary tables, TableS2b-PCR primers tab):
S6_ID - locus tag of *M. gallisepticum S6* to which the primers are designed
gene_name - gene’s name according to trivial nomenclature used for bacteria, if exists
for - forward primer

rev - reverse primer

Primers are designed to have T_m from 58° to 62°C with no more than 2°C difference in T_m. T_m was calculated according to SantaLucia method (SantaLucia J. 1998. A unified view of polymer, dumbbell, and oligonucleotide DNA nearest-neighbor thermodynamics. *PNAS* 95(4):1460-1465). Amplicons length was designed to be 300-310 b.p. on average. PCR was performed according to the following protocol: 96°C for 15 sec, 58°C for 20 sec, 65°C for 1 min for 40 cycles with 5 pmol of each primer per 21 mcl of reaction mixture.

Table S3. Transcription intervals

see file Supplementary tables, TableS3-intervals tab

File contains information about coverage steps (transcription intervals). It consists of following columns, from left to right:

pos: genome position of border (leftmost position of corresponding interval)

cov: average coverage by all samples from DS2

dir: direction of coverage step at given position (**u**p or **d**own)

strand: strand of interval

has.gene: whether given interval overlap gene by more than 50% of gene length

operon_id: intervals with the same operon id belong to single operon. Interval is not expressed if operon_id is NA

is.primary: whether this border is first or last border of operon

term.pos: genome position of nearest hairpin

tss.pos: genome position of nearest 5'-ERS-identified TSS

Table S4. TSS position (5-ERS)

see file Supplementary tables, TableS4-TSSs tab

File contains information about 5'-ERS-identified TSS. It consists of following columns, from left to right:

pos: genome position of TSS

strand: strand of TSS

spacer length: length of spacer between -10 box and TSS

gene_id: id of neares gene

tataat.seq: sequence of -10 box

Number of reads that mapped to each TSS in each sample are shown in remaining four columns.

Table S5. Analysis of differential expression of CDS under different conditions

see file Supplementary tables, TableS5-diffexpression tab

For each CDS and condition BH-corrected p-value and log2 fold change is shown. Cluster name is shown in the last column for each heat shock related CDS.

Table S6. GO-enrichment of stress-related genes

Significantly enriched terms (qv (BH-corrected p-value) is below 0.2) for each stress and direction of change are listed in table below. Only contitions/change directions that have at least one significant term are shown. Number of genes in given term and number of them that change expression significantly in given direction and under given condition are shown in “size” and “sel. size” columns respectively

**h2o2 up**					
category	qv.over	ontology	term	size	sel. size
GO:0004030	0.03	MF	aldehyde dehydrogenase [NAD(P)+] activity	4	3
GO:0006081	0.0094	BP	cellular aldehyde metabolic process	4	3
GO:0006084	0.0094	BP	acetyl-CoA metabolic process	4	3
GO:0016226	0.0094	BP	iron-sulfur cluster assembly	4	3
GO:0055114	0.021	BP	oxidation-reduction process	37	7
**h2o2 down**					
GO:0005694	0.19	CC	chromosome	7	2
GO:0045261	0.19	CC	proton-transporting ATP synthase complex, catalytic core F(1)	9	2
**nacl up**					
GO:0004030	0.078	MF	aldehyde dehydrogenase [NAD(P)+] activity	4	3
GO:0006081	0.037	BP	cellular aldehyde metabolic process	4	3
GO:0006084	0.037	BP	acetyl-CoA metabolic process	4	3
GO:0004519	0.18	MF	endonuclease activity	6	3
GO:0006950	0.19	BP	response to stress	7	3
**stat. up**					
GO:0006950	0.00004	BP	response to stress	7	5
GO:0055114	0.0036	BP	oxidation-reduction process	37	7
GO:0006096	0.0099	BP	glycolysis	12	4
**stat. down**					
GO:0016021	0.07	CC	integral component of membrane	49	48
**hs5 up**					
GO:0004252	0.0027	MF	serine-type endopeptidase activity	8	6
**hs5 down**					
GO:0016773	0.066	MF	phosphotransferase activity, alcohol group as acceptor	3	3
**hs15 up**					
GO:0004803	0.041	MF	transposase activity	24	13
GO:0006313	0.044	BP	transposition, DNA-mediated	24	13
GO:0003677	0.041	MF	DNA binding	77	29
GO:0055085	0.044	BP	transmembrane transport	12	8

GO:0042626	0.042	MF	ATPase activity, coupled to transmembrane movement of substances	10	7
GO:0004252	0.042	MF	serine-type endopeptidase activity	8	6
GO:0008982	0.15	MF	protein-N(PI)-phosphohistidine-sugar phosphotransferase activity	5	4
GO:0006304	0.19	BP	DNA modification	3	3
GO:0008643	0.19	BP	carbohydrate transport	3	3
**hs15 down**					
GO:0019843	0.07	MF	rRNA binding	33	16
GO:0003676	0.07	MF	nucleic acid binding	10	7
GO:0003735	0.07	MF	structural constituent of ribosome	51	21
**hs30 up**					
GO:0055085	0.069	BP	transmembrane transport	12	8
GO:0042626	0.07	MF	ATPase activity, coupled to transmembrane movement of substances	10	7
GO:0004252	0.07	MF	serine-type endopeptidase activity	8	6
GO:0003677	0.17	MF	DNA binding	77	26
GO:0008982	0.17	MF	protein-N(PI)-phosphohistidine-sugar phosphotransferase activity	5	4
**hs30 down**					
GO:0006412	0.043	BP	translation	53	25
GO:0003735	0.056	MF	structural constituent of ribosome	51	24
GO:0019843	0.056	MF	rRNA binding	33	17
GO:0006096	0.13	BP	glycolysis	12	8
GO:0005840	0.1	CC	ribosome	40	18

Table S7. GO-enrichment for clusters of heat shock related genes

Significantly enriched terms (qv (BH-corrected p-value) is below 0.2) for each cluster are listed in table below. Only clusters that have at least one significant term are shown. Number of genes in given term and number of them from given cluster are shown in “size” and “sel. size” columns respectively

**c1**					
category	qv.over	ontology	term	size	sel. size
GO:0006412	0.0000037	BP	translation	53	25
GO:0003735	0.0000084	MF	structural constituent of ribosome	51	24
GO:0019843	0.000088	MF	rRNA binding	33	17
GO:0005840	0.00019	CC	ribosome	40	18
GO:0006096	0.0048	BP	glycolysis	12	8
**c2**					
GO:0004803	0.00017	MF	transposase activity	24	12
GO:0006313	0.00016	BP	transposition, DNA-mediated	24	12

GO:0004252	0.029	MF	serine-type endopeptidase activity	8	5
**c3**					
GO:0055085	0.054	BP	transmembrane transport	12	6
GO:0008643	0.054	BP	carbohydrate transport	3	3
GO:0042626	0.11	MF	ATPase activity, coupled to transmembrane movement of substances	10	5
GO:0003887	0.11	MF	DNA-directed DNA polymerase activity	7	4
GO:0004030	0.11	MF	aldehyde dehydrogenase [NAD(P)+] activity	4	3
GO:0006081	0.13	BP	cellular aldehyde metabolic process	4	3
GO:0008408	0.11	MF	3'-5' exonuclease activity	4	3
**c4**					
GO:0016773	0.0087	MF	phosphotransferase activity, alcohol group as acceptor	3	3
GO:0005525	0.05	MF	GTP binding	19	5
GO:0003924	0.05	MF	GTPase activity	12	4
GO:0006184	0.14	BP	GTP catabolic process	12	4
GO:0016310	0.18	BP	phosphorylation	24	5
**c9**					
GO:0003735	0.012	MF	structural constituent of ribosome	51	6
GO:0005840	0.0075	CC	ribosome	40	5
GO:0006412	0.14	BP	translation	53	5

Table S8. CIRCE matches

CIRCE elements identified in *M. gallisepticum S6* genome with 2 or less mismatches.

ID_S6	start	end	seq	gene name	distance to CDS	distance to -10 box
consensus			TTAGCACTCNNNNNNNNNGAGTGCTAA			
Site_9	188835	188861	TTATCACTTTAGCTCTTAGAGTGCTAA	dnaJ_2	61	overlap by 3 bp
Site_10	194321	194347	TTATCACTTTAGCTCTTAGAGTGCTAA	GCW_ 90867	324	overlap by 3 bp
Site_11	494450	494476	TTAGCACTTGAATTATTAGAGTGCTAA	lon	62	13
Site_12	662204	662230	TTATCACTCTAATAGCCTAAGTGCTAA	clpB	43	8
Site_13	732791	732817	TTAGCAATCTACTTGCAAAAGTGCTAA	dnaK	92	8

Table S9. GO-enrichment for TSS and TTs heat shock up-regulated genes regulated genes

Significantly enriched terms (qv (BH-corrected p-value) is below 0.2) for TSS- and TT-regulated genes that significantly up-regulated after 30 min of heat shock. Number of genes in given term and number of them from given cluster are shown in “size” and “sel. size” columns respectively

**Terminator regulated genes**					
category	qv.over	ontology	term	size	sel. size
GO:0055085	0.021	BP	transmembrane transport	12	6
GO:0008643	0.026	BP	carbohydrate transport	3	3
GO:0042626	0.075	MF	ATPase activity, coupled to transmembrane movement of substances	10	5
**TSS regulated genes**					
GO:0004252	0.0056	MF	serine-type endopeptidase activity	8	6
GO:0004803	0.051	MF	transposase activity	24	9
GO:0006313	0.095	BP	transposition, DNA-mediated	24	9
GO:0006950	0.12	BP	response to stress	7	4
GO:0006508	0.12	BP	proteolysis	24	8
GO:0004030	0.16	MF	aldehyde dehydrogenase [NAD(P)+] activity	4	3
GO:0006081	0.12	BP	cellular aldehyde metabolic process	4	3
GO:0005351	0.16	MF	sugar:hydrogen symporter activity	8	4
GO:0009401	0.15	BP	phosphoenolpyruvate-dependent sugar phosphotransferase system	8	4
GO:0051082	0.16	MF	unfolded protein binding	8	4
GO:0008982	0.19	MF	protein-N(PI)-phosphohistidine-sugar phosphotransferase activity	5	3

Table S10. PWM of promoter sequence

see file Supplementary tables, TableS10-TSS PWM tab

Matrix contains weights (log2 of ratio of observed and background (genome-wide) nt frequencies) for each position (columns) and each of four nucleotides.

Table S11. CDS read counts

see file Supplementary tables, TableS11-CDS read count tab

Table S12. Protein abundance indexes

see file Supplementary tables, TableS12-PAI tab

Table S13. Analysis of RNA to protein correlation within GO groups

Only GO terms with at least 10 genes are shown.

term	descriptioin	gene count	control	hs5	hs15	hs30	p-value(control-hs30)
GO:0000049	tRNA binding	15	0.493	0.52	0.596	0.561	0.8164
GO:0000287	magnesium ion binding	17	0.662	0.605	0.715	0.631	0.8903
GO:0003677	DNA binding	64	0.425	0.456	0.201	0.125	0.0698
GO:0003723	RNA binding	17	0.525	0.664	0.4	0.443	0.7762
GO:0003735	structural constituent of ribosome	49	-0.079	0.075	0.065	-0.001	0.7088
GO:0003924	GTPase activity	10	0.875	0.612	0.648	0.729	0.4233
GO:0004803	transposase activity	20	0.665	0.718	0.828	0.801	0.3797
GO:0005515	protein binding	11	0.555	0.573	0.418	0.023	0.2285
GO:0005524	ATP binding	111	0.566	0.484	0.383	0.397	0.1051
GO:0005525	GTP binding	17	0.746	0.357	0.408	0.329	0.1004
GO:0005737	cytoplasm	120	0.701	0.594	0.477	0.444	0.0027
GO:0005840	ribosome	37	0.077	-0.016	0.035	-0.03	0.6588
GO:0005886	plasma membrane	34	0.62	0.453	0.569	0.692	0.6212
GO:0006096	glycolysis	12	0.326	0.657	0.683	0.566	0.5187
GO:0006184	GTP catabolic process	10	0.875	0.612	0.648	0.729	0.4233
GO:0006200	ATP catabolic process	20	0.474	0.528	0.584	0.512	0.8814
GO:0006260	DNA replication	21	0.732	0.675	0.684	0.243	0.0395
GO:0006313	transposition, DNA-mediated	20	0.665	0.718	0.828	0.801	0.3797
GO:0006412	translation	51	-0.041	0.017	0.038	-0.031	0.9593
GO:0006457	protein folding	12	0.872	0.755	0.399	0.406	0.0531
GO:0006508	proteolysis	18	0.51	-0.143	0.205	0.135	0.2426
GO:0006810	transport	14	0.297	0.479	0.711	0.528	0.51
GO:0008152	metabolic process	14	0.763	0.774	0.56	0.516	0.3122
GO:0008270	zinc ion binding	19	0.391	0.442	0.222	0.168	0.4913
GO:0016020	membrane	17	0.273	0.199	0.347	0.108	0.6504
GO:0016021	integral component of membrane	25	0.516	0.482	0.623	0.641	0.5318
GO:0016310	phosphorylation	21	0.879	0.725	0.705	0.668	0.0897
GO:0016740	transferase activity	10	0.527	0.612	0.212	0.37	0.7107

GO:0016887	ATPase activity	17	0.559	0.62	0.651	0.554	0.985
GO:0019843	rRNA binding	33	0.08	0.258	0.15	0.146	0.794
GO:0046872	metal ion binding	28	0.706	0.581	0.6	0.698	0.9574
GO:0055114	oxidation-reduction process	33	0.88	0.773	0.669	0.537	0.0027
GO:0090305	nucleic acid phosphodiester bond hydrolysis	19	0.544	0.525	0.417	0.109	0.1573

Table S14. RT-PCR analysis of heat shock-related regulation of four selected hairpinless down-CS

Normalized threshold cycle for three controls (C-1, 2 and 3) and three heat stresses (HS30-1, 2, and 3) samples. Two pairs of primers were designed for each down-CS: upstream of down-CS (Up) and downstream of down-CS (down). Used primers are listed at the bottom of primer table of supplementaryTableS2.PCR.xls.

Step ID	C-1	C-1	HS30-1	HS30-1	C-2	C-2	HS30-2	HS30-2	C-3	C-3	HS30-3	HS30-3
	Up	Dn	Up	Dn	Up	Dn	Up	Dn	Up	Dn	Up	Dn
8	25.73	29.26	25.4	26.12	25.26	27.78	23.98	24.29	26.35	29.24	25.3	25.71
19	23.23	25.75	25.06	25.58	23.12	25.13	24.23	24.85	24.06	25.72	25.44	26.17
119	23.22	24.2	24.41	24.05	22.21	22.42	22.67	21.76	23.16	23.31	23.81	23.16
1008	24.02	23.46	23.22	22.62	24.3	23.38	22.1	20.3	23.94	24.06	23.11	22.13

Table S15. Transformation frequencies with different vector amount.

Plasmid amount	CFU per 10^9 cells (1 ml of late-logarithm culture)	Transformation efficiency
10 μg	10^3	10^{-06}
1 μg	10^3	10^{-06}
100 ng	10^3	10^{-06}
10 ng	$2*10^2$	$2*10^{-07}$
1 ng	30-50	$2*10^{-08}$

Table S16. Positions of vector integration sites.

Clone	Position	Locus
clone-1	931568	Intergenic region
clone-2	960197	GCW_03980 - hypothetical protein
clone-4	242561	GCW_01075 - *spoT,* ppGpp synthase

Table S17. Transcription of *tetM* gene in transformants by RT-qPCR.

ND - not detected

	clone-1	clone-2	clone-3	clone-4
transposase rep1	ND	ND	ND	ND
transposase rep2	ND	ND	ND	ND
tetM rep1	22,38	17,18	16,48	18,96
tetM rep2	22,52	17,47	16,68	19,14
gapd rep1	22,48	20,24	20,36	20,33
gapd rep2	22,79	20,64	20,60	20,75
eno rep1	23,67	21,86	22,76	21,85
eno rep2	23,37	22,18	22,02	21,73

Supplementary Figures

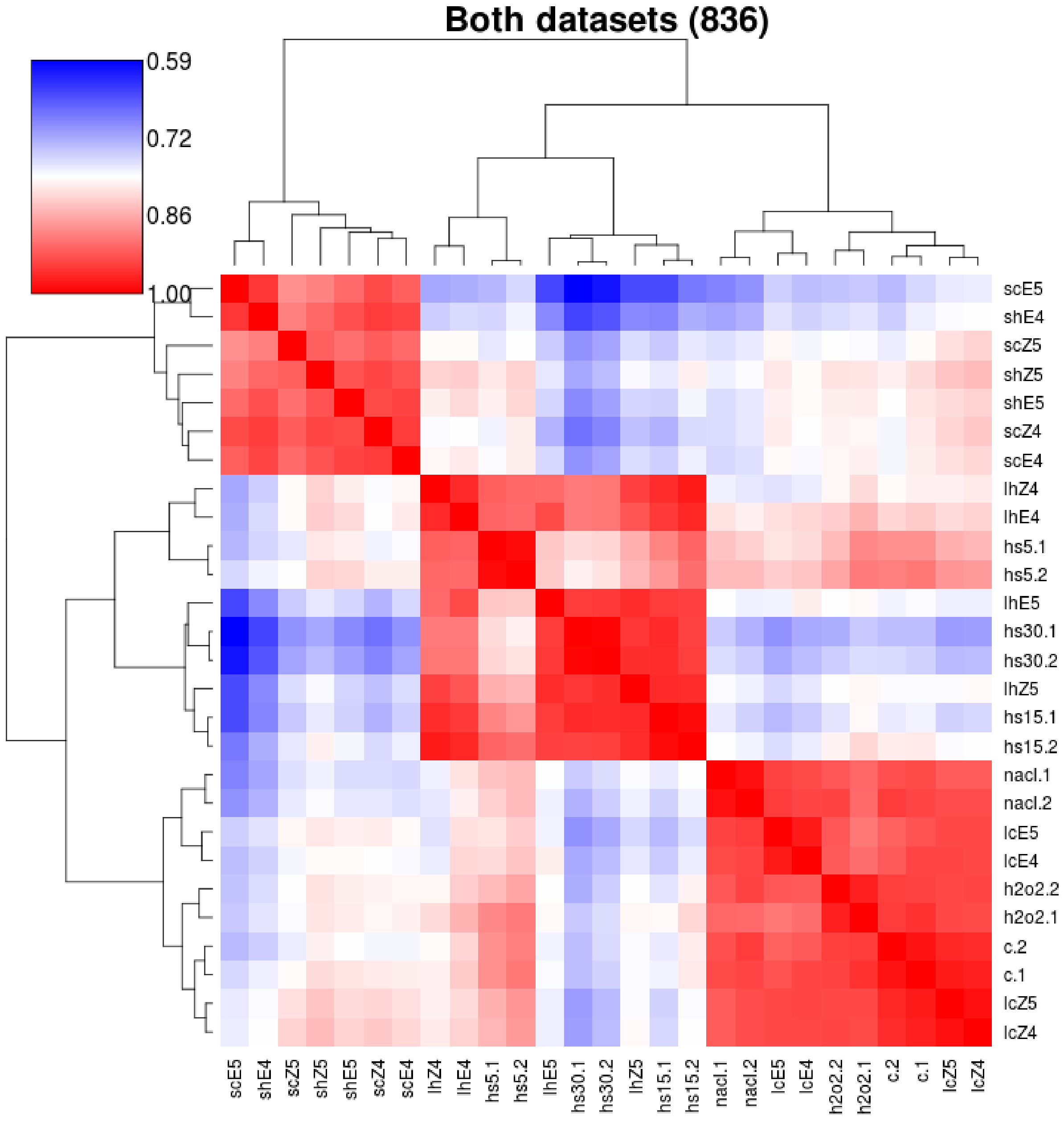

Figure S1. Correlation heatmap for all RNA-Seq samples

Spearman correlation of CDS read counts for all RNA-Seq samples is shown by heatmap. Samples were reordered by hierarchical clustering using complete linkage and 1-cor as distance.

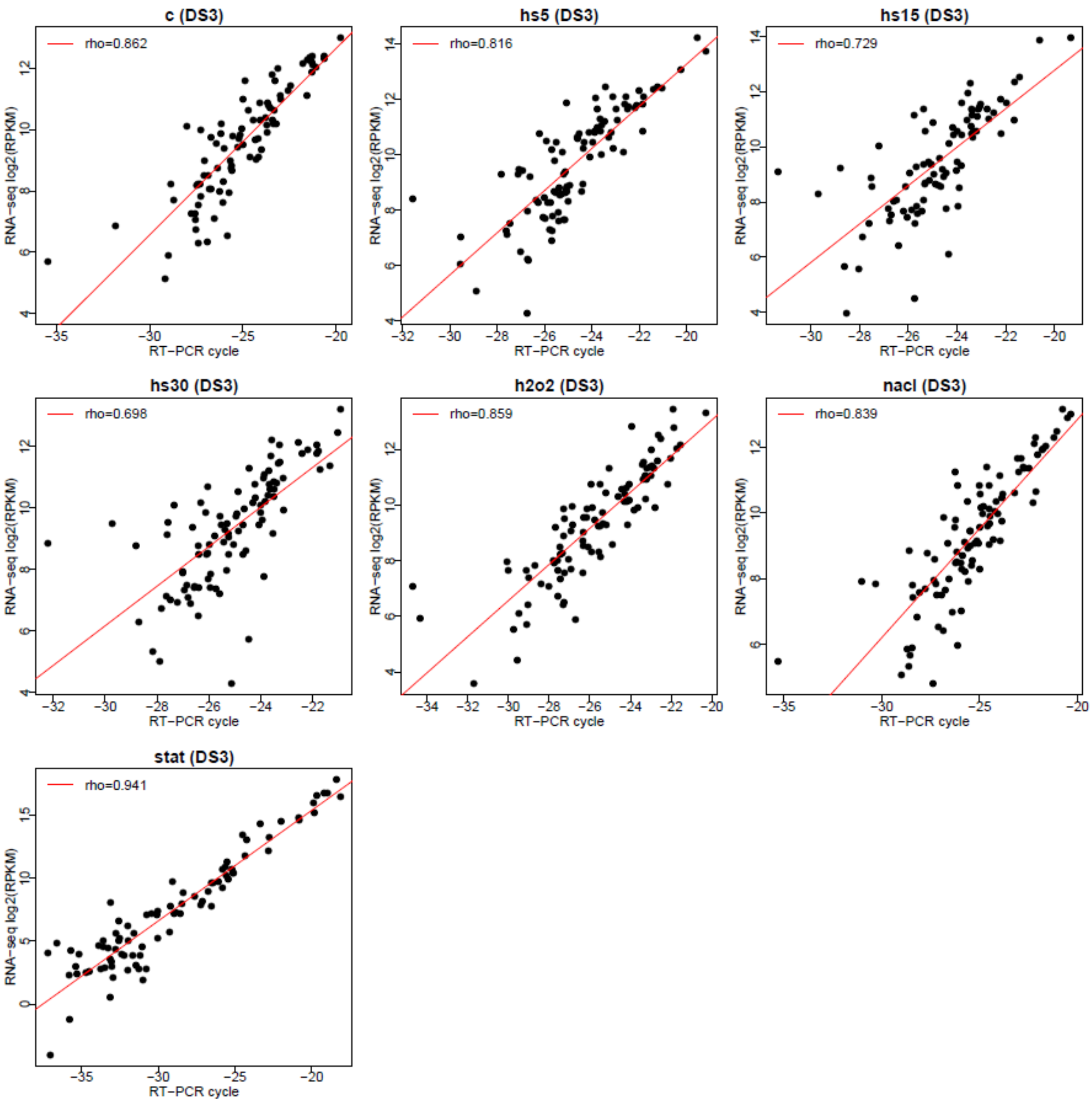
c (DS3)
rho=0.862
hs5 (DS3)
rho=0.816
hs15 (DS3)
rho=0.729
hs30 (DS3)
rho=0.698
h2o2 (DS3)
rho=0.859
nacl (DS3)
rho=0.839
stat (DS3)
rho=0.941
RNA−seq log2(RPKM)
RT−PCR cycle

Figure S2. Correlation between RT-PCR- and RNA-Seq-based gene expression estimates

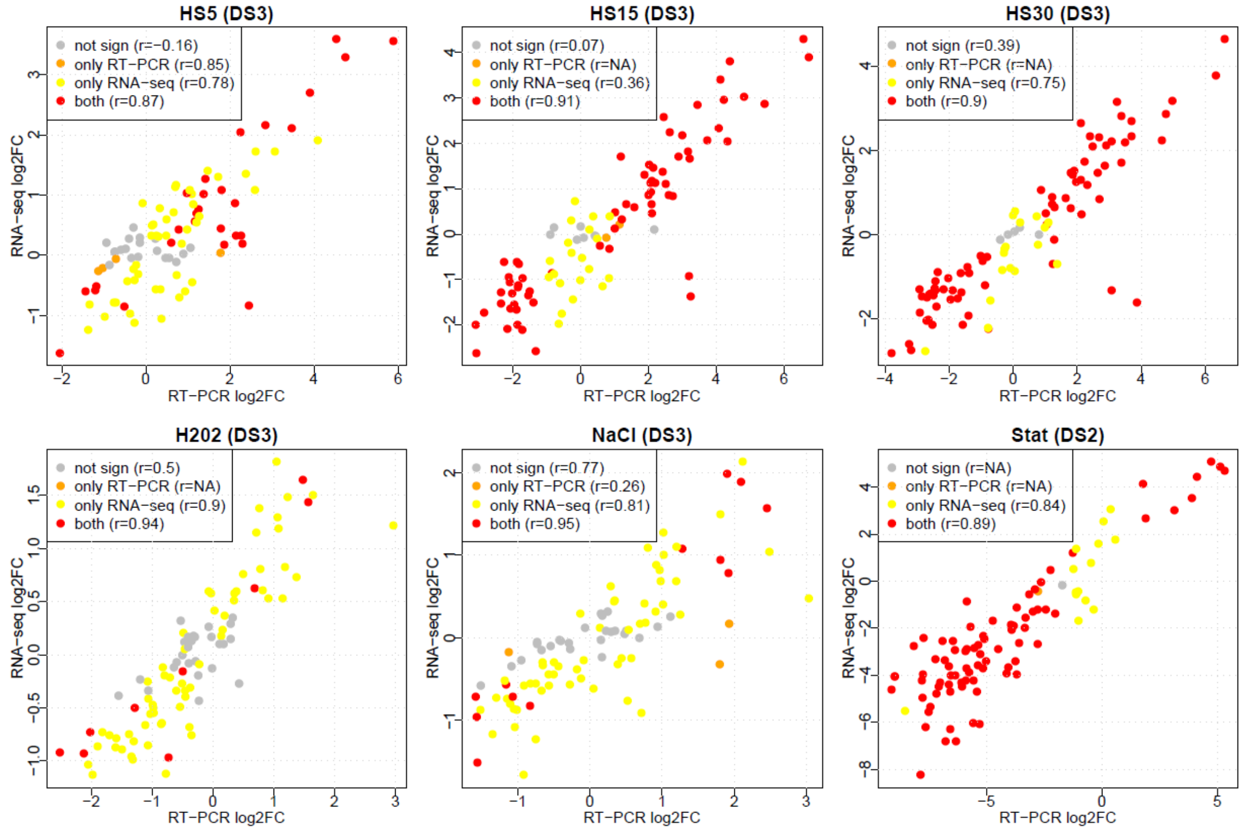

Figure S3. Correlation between RT-PCR- and RNA-Seq-based gene expression fold change estimates

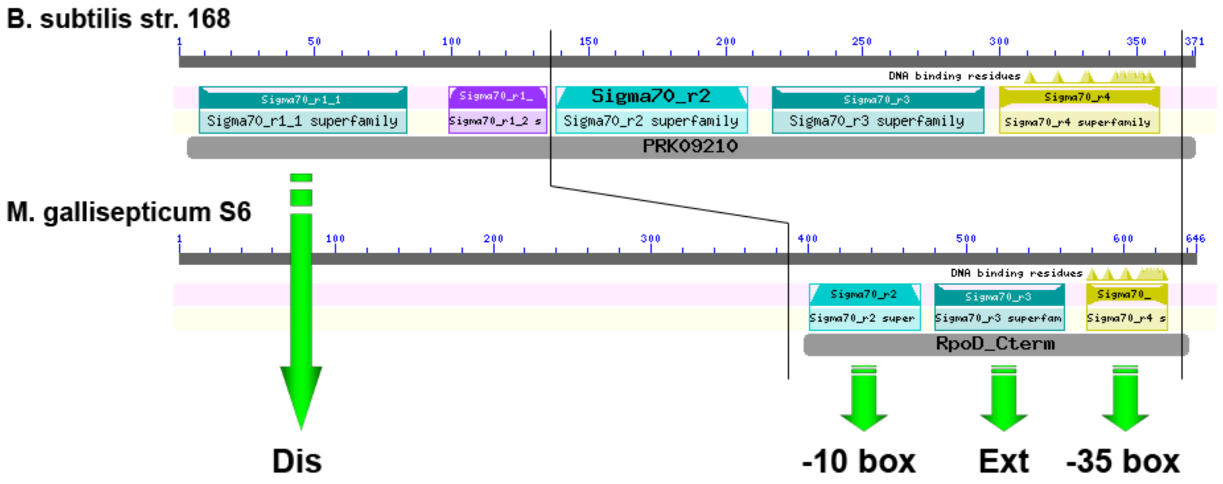

Figure S4. Sigma factor structure

Structure of *M. gallisepticum S6* main sigma factor *rpoD* in comparison with *B. subtilis subsp. subtilis str 168* main sigma factor *rpoD* (*sigA*). Domains are identified by NCBI CDD. N-terminal fragment of sigma factor is replaced in *M. gallisepticum* with a sequence of an unknown function. Discriminator-binding region is absent. C-terminal sequence with -10 and -35 binding regions is present in *M. gallisepticum*.

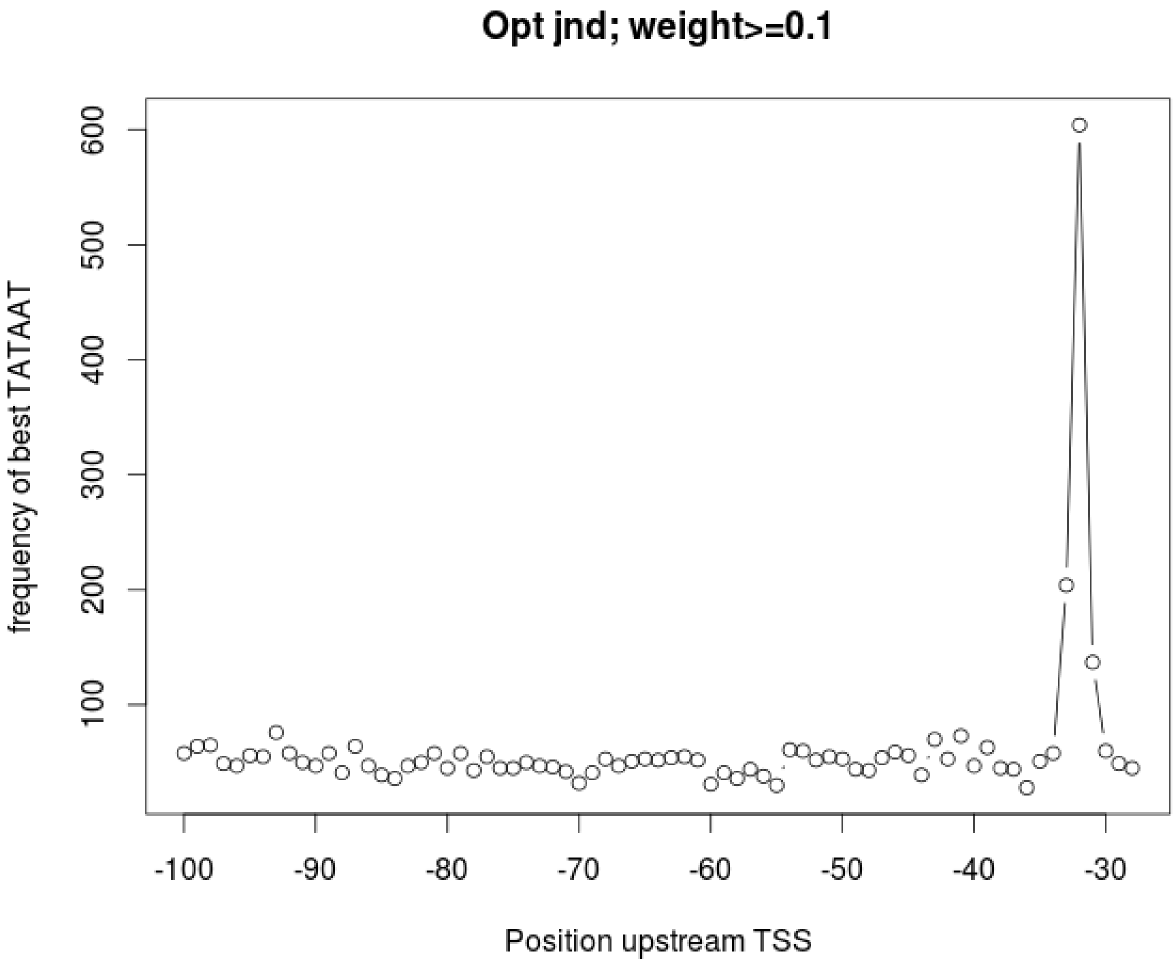

Figure S5. Distribution of best match of promoter PWM in 100nt upstream of TSSs

Distribution of position of a best match with weight above 0.1 (averaged by all PWM positions) for promoter PWM in 100nt upstreams of 32148 possible TSS (peaks of read coverage detected in at least one 5’-ERS sample).

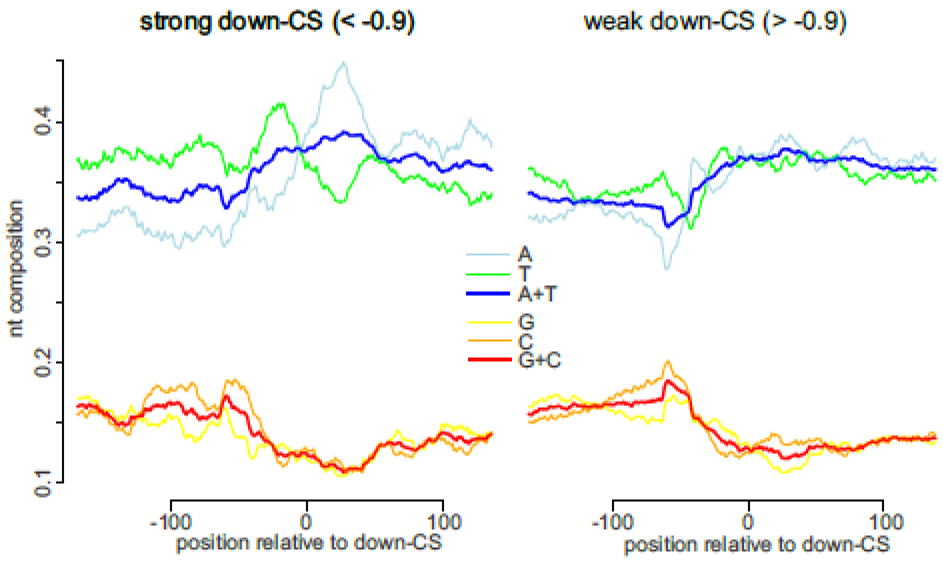

Figure S6. AT-content near down-CS.

Average nt-content of +-150nt region around strong (relative step size below -0.9, left) and weak (relative step size above -0.9, right) down-CS

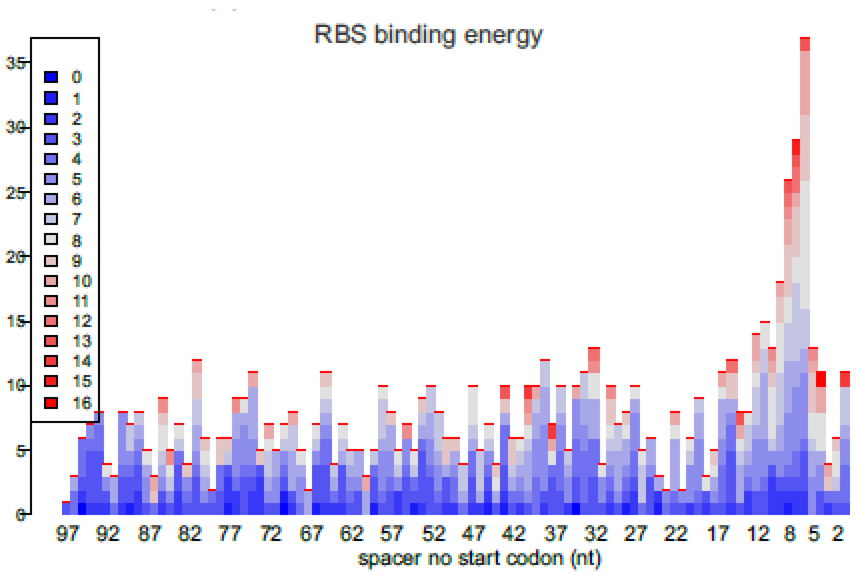

Figure S7. Ribosome binding energy

Distribution of best RBS by free energy (in -kcal/mol) of duplex (color) and position relative to start codon.

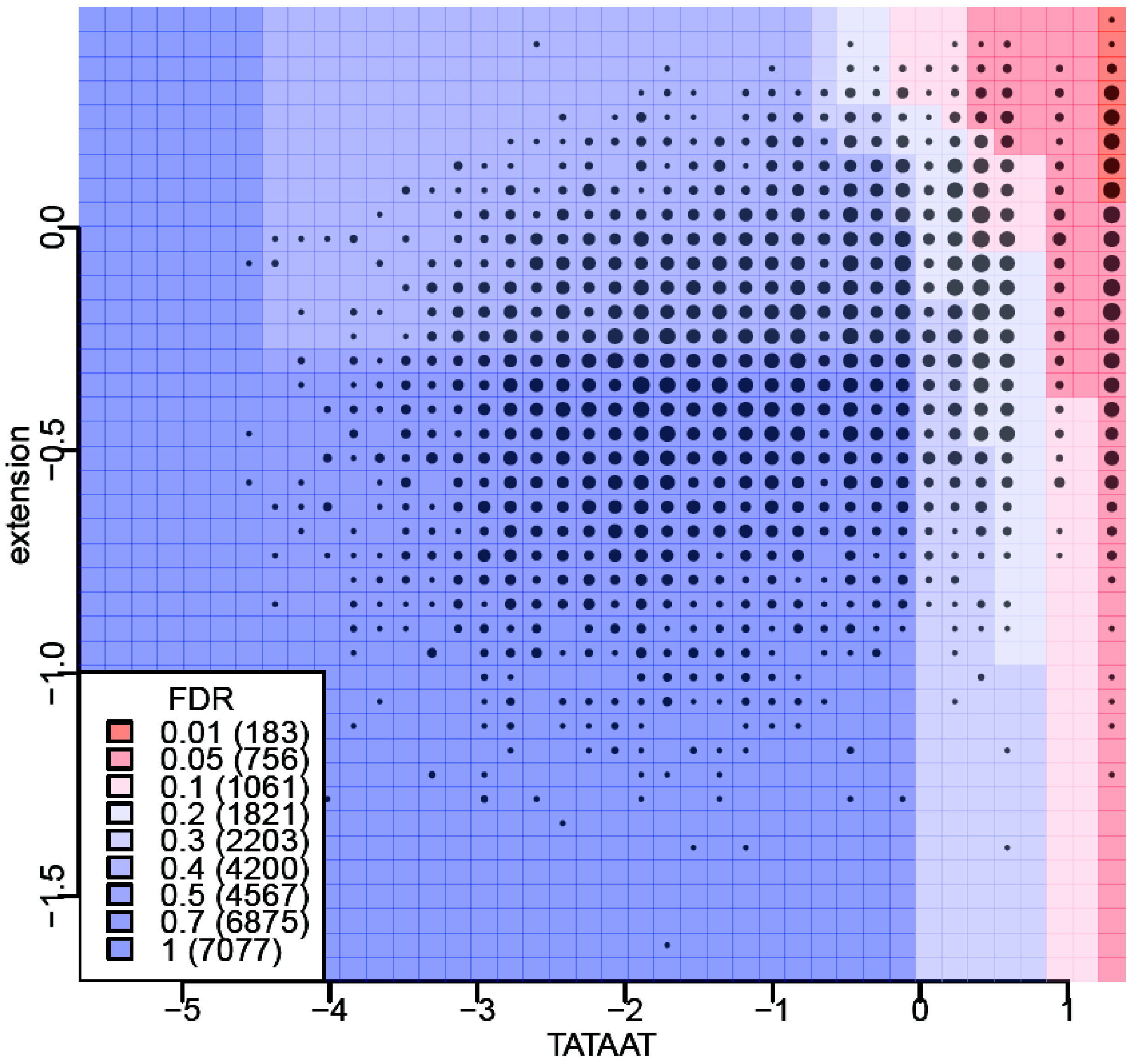

Figure S8. Dependence of FDR of -10 box and extension weights

Distribution of 7077 possible TSS, that were detected either in two control 5'-ERS samples or in two heat shock samples or both, by PWM weight of -10 box and it's extension (20 nt upstream and 3 nt downstream of -10 box) sequence. Sizes of point are proportional to log of number of TSS in corresponding bin. Dependence of FDR on weight of extension is shown by color in each column. Numbers of TSS with FDR below given cutoff are listed in legend.

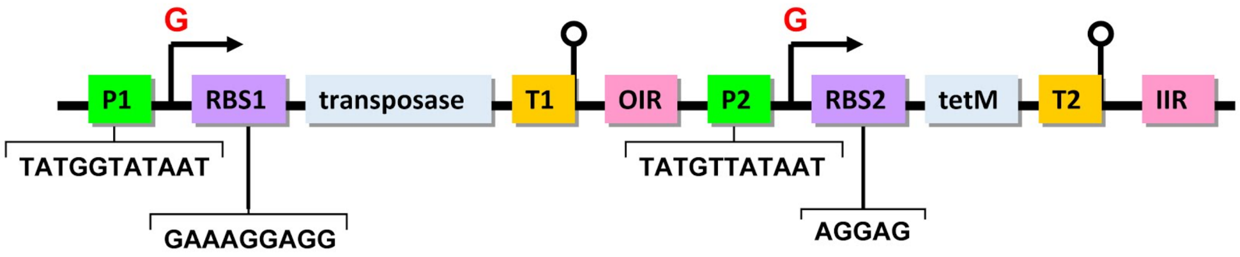

Figure S9. Vector design for *M. gallisepticum* transformation

P1, P2 - promoters, RBS1, RBS2 - ribosome binding sites, T1, T2 - intrinsic terminators (T1 is rRNA precursor terminator, T2 is *dps* terminator), transposase - Tn4001 transposase gene, tetM - tetracycline resistance gene, OIR and IIR - inverted repeats required for transposition. Red letter above TSS position shows first nucleotide of the transcript. For sequence and detailed annotation see supplementary file supplementaryFile.vector.gb.

```
rpsP--------GGCACTGAACAATCAGTTAAGTAATTTTACT-31
tetM--------ATTCCTGAACAATCAGTTAAGTAATTTTACT-31
transposase-------------ATATTCTTCTTTAAGAAAT-19

rpsP--------TAACGATATGTTATAATATTTCAGTTAGAAA-62
tetM--------TAATGATATGTTATAATATTTCAGTTAGAAA-62
transposase-AATTAATATGGTATAATATTTTAGTTAGAAA-50

rpsP--------AATTT--AAGGACA-AATAAC-------ATG-83
tetM--------AATTT--AAGGAGA-AATAACGGATCC-ATG-89
transposase-AATTTGAAAGGAGGTAATAACAAGATCTATG-81
```

Figure S10. Alignment of upstream regions of *rpsP* (prototype), *tetM* and transposase.

Yellow highlights core promoter (including Ext-element, -35 box is absent), blue highlights RBS, italic shows initiator nucleotide, start codon is shown in bold. Optimized nucleotides are underlined.

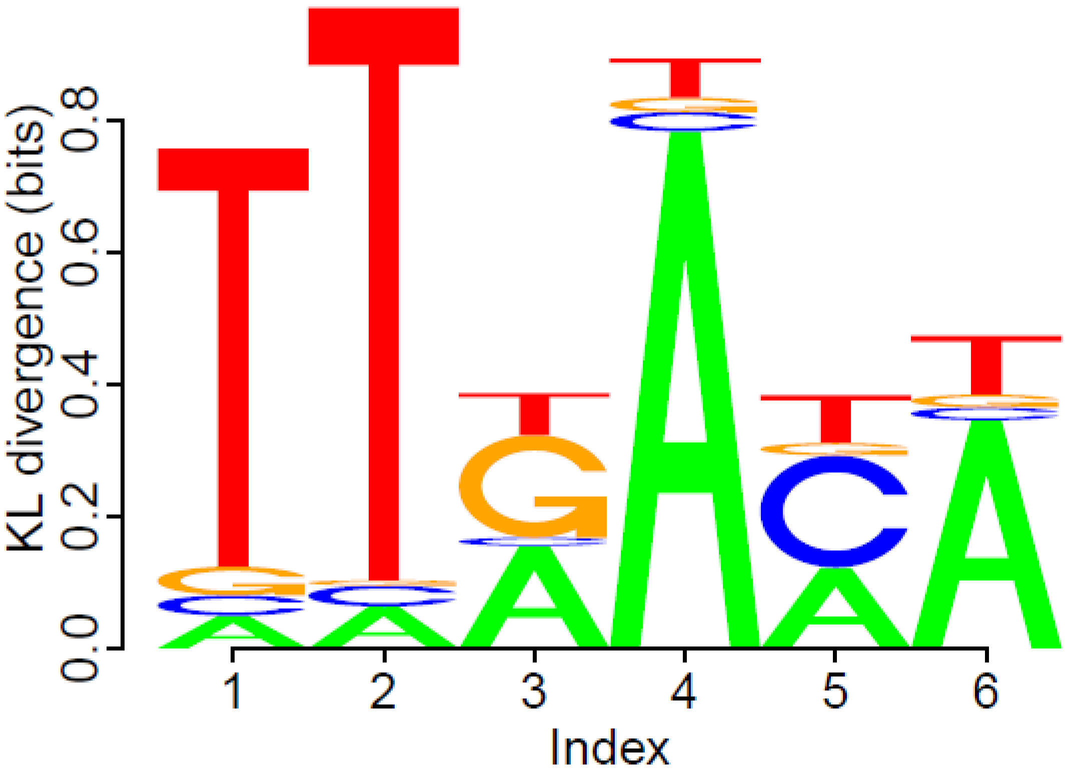

Figure S11. Logo-image of -35 box

122 promoters that have TTGACA sequence with not more than two mismatches was used to plot the figure.

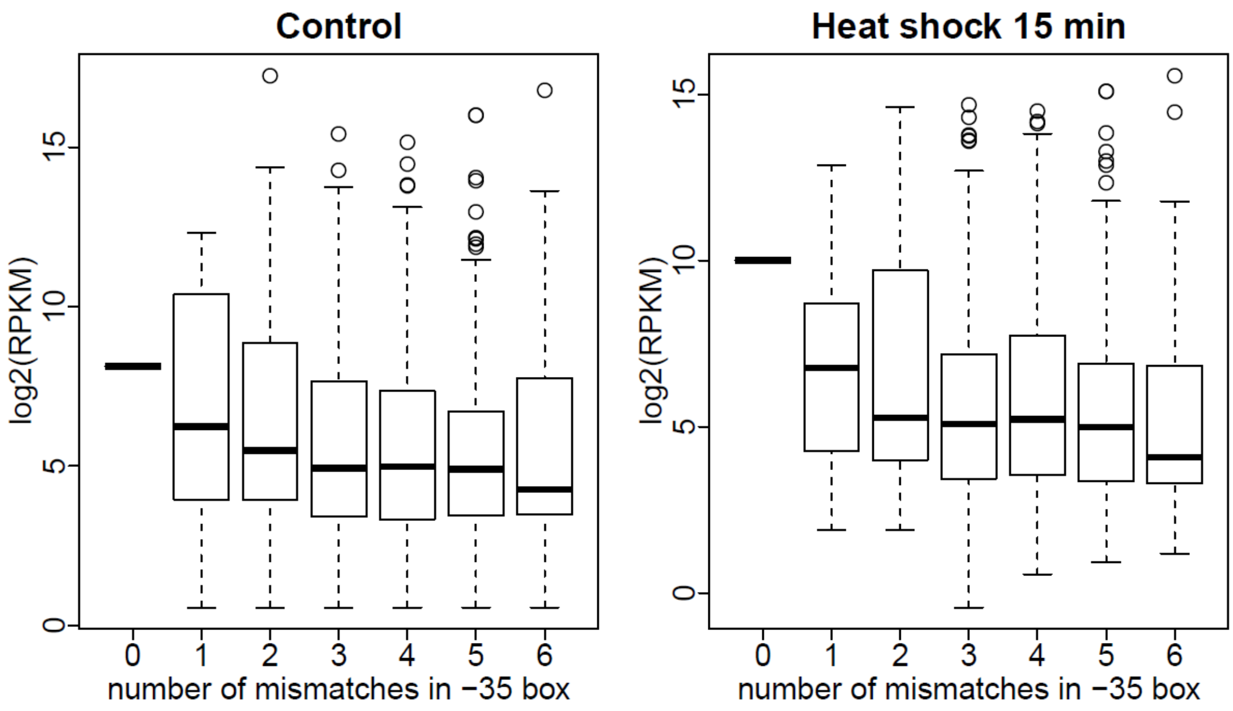

Figure S12. Dependence of TSS activity on number of mismatches in -35 box compared with consensus sequence (TTGACA)

TSSs with less than three mismatches have significantly greater activity than others (Two-sided Wilcoxon test, pv < 0.004 and 0.002 for control conditions and heat shock respectively).
